# Book Review

**Published:** 1906-06

**Authors:** 


**Dispensing Made Easy.** By Wm. G. Sutherland, M.B. Second Edition. Pp. vii., 104.—Bristol: John Wright & Co. 1905.—This book has been compiled as an aid to medical men in compounding medicine for their patients. It opens with useful advice regarding the fitting up and arrangement of a dispensary. The author apparently advocates the practice of "dotting poisons here and there," rather than of keeping them together in one cupboard—very doubtful advice. He then proceeds to give hints, and some very good ones, on the preparation of various medicaments. Some of his remarks lead us to think that he regards his brothers as lacking in common sense. For instance, to remove broken corks from inside bottles, "invert the bottle, and endeavour to noose the cork with stiff string or wire"—quite a sporting method. Mr. Sutherland then explains how the drug bill may be cut down, quoting certain figures in support of his remarks. The book is largely occupied by formulæ for numerous mixtures, those for parish practice being so simple that "they may be safely dispensed by any intelligent nurse with a month's training." We fail to see why simple mixtures can safely be dispensed by novices, especially in a dispensary where the system of scattering poisons prevails. Time only will prove the value of such a text-book to those engaged in practices where a sound knowledge of *extempore* pharmacy is requisite.

**The Theory and Practice of Medicine.** By FREDERICK J. ROBERTS, M.D. Tenth Edition. 2 volumes. Pp. xiii., 1,368. London: H. K. Lewis. 1905.—Since the last edition of this work many advances have been made in medical science. These the author has, as far as possible, incorporated in his book, bringing it thoroughly up to date. That it is not a compilation, but embodies the experiences and personal opinions of an author after many years' professional work, adds much to the value of the book. Much of the more recent work on the organisms connected with tropical diseases has been added. The articles on the diseases of the skin have been omitted, as the author considers the subject has become too extensive to be adequately treated in a text-book on general medicine. As a text-book the work can be highly recommended, and that it has reached the tenth edition proves that it is deservedly popular.

**Clinical Methods.** By ROBERT HUTCHISON, M.D., and HARRY RAINY. Third Edition. Pp. xii., 634. London: Cassell and Company, Limited. 1905.—This well-known manual has been thoroughly revised and brought up to date. Since the last edition many important advances have been made in the realm of clinical pathology, and those which are likely to be of practical value at the bedside have been added to the book. The subject is one which is growing daily in importance and extent. The descriptions are clear, concise, and accurate, and a considerable amount of useful information is obtained in a small compass. The book is undoubtedly one of the best which can be owned by a medical student.

**The Nervous Affections of the Heart.** Being the Morison Lectures delivered before the Royal College of Physicians of Edinburgh in 1902 and 1903. By GEORGE ALEXANDER GIBSON, M.D. Pp. 99. Edinburgh: Young J. Pentland. 1904.—These very interesting and well-written lectures were delivered before the Royal College of Physicians of Edinburgh in 1902 and 1903, and published in the *Edinburgh Medical Journal*. We are glad to have them in this more convenient form, in which they will be accessible to a larger number of readers. They are divided into two sections: the first dealing with the sensory, and the second with motor disturbances of the heart. We can recommend the book to the perusal of our readers as presenting an adequate and lucid account of a subject of great interest and practical importance on which much work has recently been done. The lecture on the therapeutics of sensory cardiac disorders will be found a very useful one.

**Drink Restriction (Thirst Cures) Particularly in Obesity.** Being Part VI. of *Diseases of Metabolism and Nutrition.*

By Professor C. von Noorden and Dr. Hugo Salomon. Edited by Boardman Reed, M.D. Pp. 86. Bristol: John Wright & Co. 1905.—This is an interesting and useful little book. It gives a short historical sketch of the therapeutic employment of restriction of fluid, which shows that this method of treatment was to some extent employed by the ancients and during the Middle Ages. There is an account of physiological investigations into the effects of thirst upon the metabolic processes in the organism, and of some recent experiments of the authors. The latter were made on persons who were (1) obese and (2) not obese, and show that loss of fat is not a primary consequence of restricting liquids, and that the statements of Oertel and Schweninger are misleading in this respect. If loss of fat occurs "it must be regarded as dependent on certain concomitant factors and influences, chief among them, undoubtedly, the loss of appetite that occurs when the patient is made to suffer thirst." The large fall in weight that occurs in the initial period of a "thirst cure" in the obese is due to loss of water by the organs and tissues. Increased destruction of proteid tissues with increased nitrogenous output can be obtained by restricting the amount of fluid taken, but only when it is restricted to such an extent as to produce torturing thirst, carried, that is, beyond the limits of therapeutic procedure. Deprivation of fluid, especially at meal times, in many persons means loss of appetite, and hence indirectly loss of weight, but this loss of appetite is by no means an invariable result. Restriction of fluid is valuable in heart disease and in other disorders of the circulatory apparatus, because it lessens the total work to be performed by the latter on account of the diminished volume of the blood. In obese subjects with feeble heart deprivation of fluid, which the author does not carry beyond 1½ litres per diem, irrespective of the water in the food, is therefore useful. There is some evidence that fluid restriction is valuable in selected cases of chlorosis. It is also useful in dilatation of the stomach, in some other forms of dyspepsia, and at certain stages in the course of acute nephritis and of contracted granular kidney. The reader will find in the book much useful information as to the use of this simple but very powerful therapeutic measure. Some of the words used are ugly, *e.g.* "fore-period" and "practician." The dibliography at the end of the work deserves mention.

**On the Hours of Sleep at Public Schools.** By T. D. Acland, M.A., M.D. Pp. 33. London: J. and A. Churchill. 1905.—This inquiry, based on statistics obtained from forty of the great public schools in England and others in the United States of America, reveals the fact that the hours of rest in bed allotted to schoolboys in this country are considerably fewer than in America or Sweden. Dr. Acland is of opinion that at a period when new bodily and intellectual functions are rapidly unfolding, the minimum amount of rest should be from nine to nine and a half hours. The pamphlet sets forth clearly and succinctly the reasons that have led the author to this conclusion, and discusses the objections which have been raised to the extension of the hours of sleep. Parents and teachers and medical officers of schools are much indebted to Dr. Acland for this valuable little monograph.

**The Medical Society's Transactions.** Vol. xxviii. London: Harrison & Sons. 1905.—The most noteworthy portion of this volume is that containing the Lettsomian Lectures, on functional mental disorders, given by Dr. G. H. Savage, whose large experience of mental diseases enables him to speak with great authority on the question. It is commonly believed that organic changes imply some visible lesion which is likely to be permanent, but Dr. Savage endeavours to show that with regard to mental diseases, disorders of function are themselves the cause of material changes which are later found in the nervous system, and that it is not to be expected that mental disorder should always be represented by a material pathological basis.

**The Nature and Treatment of Cancer.** By John A. Shaw-Mackenzie, M.D. Pp. 91. London: Baillière, Tindall & Cox. 1905.—This volume is an expanded form of the author's former work published under the title of *Some Methods of Hypodermic Medication in the Treatment of Inoperable Cancer.* He discusses the effect of Chian turpentine, oleate of soda, and trypsin given hypodermically, and claims that by their use there is an apparent arrest of growth, relief of pain, and arrest of fœtor in cases of ulceration. The possible fallacies in the conclusions formed in the treatment of cases of inoperable cancer—correctly or incorrectly diagnosed as such—are numerous, but we have so often been disappointed in the results of the so-called "cancer cures" that we must wait for the verification of the author's statements before we can decide that the treatment suggested is really beneficial.

**Materia Medica and Therapeutics.** By J. Mitchell Bruce, M.D. New [7th] Edition. Pp. x., 632. London: Cassell and Company, Limited. 1905.—Since the publication of the last edition of this well-known work nearly ten thousand copies have been sold, and this fact alone shows it has gained favour with medical men and medical students. Materia medica is often considered a dry and useless subject, but when studied in conjunction with chemistry and therapeutics becomes a most fascinating study. The author has recognised this fact, and is to be congratulated on the general arrangement of the subject matter. In the section on materia medica the physical and chemical properties of drugs are considered, and each substance is followed by a brief and accurate description of its use. The chemical formulæ used are not always quite modern, but in most cases are beyond criticism. That portion of the work dealing with therapeutics is very ably written, and the views on physiological questions are sound and up to date. Altogether we have no hesitation in recommending the work to medical students and others requiring a reliable text-book.

**The Physiology and Therapeutics of the Harrogate Waters, Baths and Climate, applied to the Treatment of Chronic Disease.** By William Bain, M.D., and Wilfrid Edgecombe, M.D. Pp. xii., 300. London: Longmans, Green & Co. 1905.—The aim of the authors is to supply evidence showing that the therapeutics of the local waters and baths rests on a secure foundation, comparable to that of drug therapeutics. The number and variety of the medicinal springs of Harrogate, which emerge within the compass of a few acres, is no less than eighty, and the various waters, showing wide differences in character, maintain their individual characteristics with great constancy. This is accounted for by the tilting of the geological strata to the surface, the springs from varying depths gaining exit between layers which are impervious. Sixteen of these waters are used internally: in the natural state the waters are athermal, but they are heated artificially before reaching the taps from which they are dispensed. The saline constituent being common to them all, they may be classified under two groups—saline-sulphur and saline-chalybeate, of varying strengths, mild, medium or strong. The sulphur occurs in the form of sodium sulphydrate, NaHS, sodium sulphide, $Na_2S$, or sulphuretted hydrogen gas, $H_2S$. This latter constituent gives the special characteristic to the water of the old sulphur well, the best known and most valuable of the Harrogate springs. The book is a reliable guide, with little padding and no puffing.

**Dental Surgery.** By A. W. Barrett, M.B., L.D.S. Fourth Edition. Pp. xii., 159. London: H. K. Lewis. 1905.—This is a useful book to those for whom it is written. Most of the information it gives would be endorsed by every dental surgeon. But we take exception to the statement on page 97 that modern opinion favours the retention of stumps. If there is one thing more than another getting more apparent every day it is the evil wrought by septic conditions of the mouth. We do not like the look of the gag illustrated on page 122.

**Index-Catalogue of the Library of the Surgeon-General's Office, United States Army.** Second Series. Vol. X., *M–Mnikhovski.* Pp. 935. Washington: Government Printing Office. 1905.—The present volume of this indispensable work includes 10,122 author titles, 10,856 subject titles, and 22,622 titles of articles in periodicals. When we consider that this is but one volume of the second series, which will probably reach eighteen volumes before it is finished, we can but marvel at the indefatigability of the librarian and his assistants, to whom the cordial thanks of the profession are due.

**Medical Laboratory Methods and Tests.** By HERBERT FRENCH, M.D. Pp. viii., 152. London: Baillière, Tindall, & Cox. 1904.—A handbook which, like the one before us, gives an able and critical *résumé* of the chief practical tests used in the investigation of the urine, blood, sputum, fæces, etc., fulfils a very useful purpose. The microscopical examination in affections of the skin, of cerebro-spinal fluids, etc., make this a most complete handbook for the busy practitioner and for the use of students in the wards of a hospital, and to such the book will be of the utmost service.

**Hygiene and Public Health.** By B. ARTHUR WHITELEGGE, C.B., M.D., B.Sc. and GEORGE NEWMAN, M.D. Tenth Edition. Pp. viii., 636. London: Cassell & Co. Ltd. 1905. —The appearance of ten editions of this manual in fifteen years is evidence alike of its usefulness to the student and of the care which the authors take in keeping it up to date. The object stated in the preface, namely to present a concise summary of the present position of public health matters for the purposes of the Medical Officer of Health and the student, seems throughout the book to be well fulfilled. As evidence of recent revision may be cited, among other things, the mention of the epidemics of cerebro-spinal meningitis in New York, of sore throat in Colchester, and of small-pox in Dewsbury, all in 1905, while such recently discussed points as Hutchinson's fish theory of leprosy, the relation of earth temperature to summer diarrhœa, and municipal milk depôts are dealt with in sufficient detail for a work of this size. Considerable care has been given to the bacteriological portions of the book, which on the whole is quite up to date; we have failed, however, to find any reference to a few points which might usefully have been inserted, such as the position of Shiga's and Flexner's bacillus as a causative factor in dysentery and the summer diarrhœa of infants, the clumping reaction in Malta fever, and condition known as paratyphoid fever, for which a strain of Gaertner's bacillus is thought to be responsible. Meteorology, the construction of houses and drains, and the disposal of sewage are adequately treated, and considerable space is devoted to diseases due to articles of food; milk among these is very fully discussed, and due stress is laid on the importance of prompt and effective cooling. The subject of so-called "sterilization" of milk and the effect of the process on both the milk and the spore-forming organisms ("peptogenic bacteria") might perhaps have been dealt with at rather more length. The chapters on specific epidemic diseases are concise but very clear, and the construction and uses of isolation hospitals are definitely and fully described. The authors do not agree with the recent views which have been expressed as to the use of scarlet fever hospitals. The chapters on the numerous acts and regulations affecting public health and the duties of medical officers are lucid and sufficiently full; among the statistical tables at the end, one showing the small comparative decrease in the infant mortality rate might usefully have been inserted. The space devoted to the various subjects seems remarkably well apportioned, and on the whole this manual may be confidently recommended to those about to enter on the study of public health.

**A Manual of Physiology.** By G. N. Stewart. Fifth Edition. London: Baillière, Tindall, and Cox. 1906.—This popular text-book is too well known to need much introduction. It is an excellent example of the type of book that should be read for the Intermediate M.B. (London) and the First F.R.C.S. It is very full and detailed, admirably in touch with modern teaching and trend of opinion, and at the same time is neither too bulky nor losing interest by becoming a mere catalogue of facts. The diagrams, too, are most instructive. The special features of the last edition are best seen in the chapters on Blood, Digestion, and that ever-changing field, the Central Nervous System. For instance, in the chapter on Blood, an account of the coagulation is introduced that those readers whose physiology is ten years old would scarcely recognise. The old "fibrinogens" and "fibrin-ferments" are brought into line with the modern conceptions of zymogens and kinases. The influence of this recent development of thought is also well seen in the account of the digestive ferments. A good summary is also given of the very valuable recent work on the agglutinins and precipitins. The theories of hearing and colour vision might perhaps have received more attention in the light of recent suggestions, especially Gray's modification of the Helmholtz piano theory.

**On Means for the Prolongation of Life.** By Sir Hermann Weber, M.D. Pp. 104. London: John Bale, Sons and Danielsson. 1906.—This is a second enlarged edition of a lecture delivered before the Royal College of Physicians in December, 1903. The author's rules for the attainment of the object in view are summarised as follows:—1. To exercise and maintain in vigour all the organs and tissues of the body by regular daily walks or rides, supplemented by breathing and gymnastic exercises, and periodic walking and climbing tours. 2. To practice moderation in eating, drinking, and all bodily enjoyment. 3. To endeavour to obtain abundance of pure air in the house, and to spend as many hours as possible in the open air. 4. To maintain and increase the resisting powers of the body, and counteract the inherited tendencies to various diseases. 5. To create as far as possible the habit of going early to bed and of rising early, and to restrict the hours of sleep in adult life to six, or seven, or exceptionally eight. 6. To promote a healthy condition of the skin by daily baths or ablutions. 7. To keep the mental faculties in regular occupation by appropriate work. 8. To cultivate sympathy, equanimity, contentedness, cheerfulness, and hopefulness, and the great power of the will towards pursuing the path of duty and controlling anger, vanity, envy, jealousy, and all other passions.

**Reports from the Laboratory of the Royal College of Physicians, Edinburgh.** Vol. IX. Edinburgh: Oliver and Boyd. 1905.—This volume contains, in many interesting papers, the records of original investigations carried out in the laboratory of the Edinburgh Royal College of Physicians from 1903 to 1905. Part I., consisting of fourteen articles, is devoted chiefly to researches by Drs. Noel Paton and Alexander Goodall on the physiology of the spleen and supra-renal glands and the pathology of the blood. The conclusions drawn from their experiments with supra-renal extract suggest that this substance when injected in sufficiently large doses produces histological changes in two ways—first by its effect upon the blood pressure, secondly by a direct toxic effect. The altered blood pressure caused intense congestion and œdema of the lungs, and the production of fluid in the peritoneal, pleural and pericardial cavities. The histological changes were found principally in the liver and kidneys, the former in many instances showing central necrosis of the lobules, while in the latter the changes varied from marked congestion with cloudy swelling to parenchymatous nephritis with desquamation of the cells in the tubules. Part II. contains thirteen articles on a variety of subjects. In a paper on the influence of diet in pregnancy upon the weight of the offspring Dr. Noel Paton refers to the possibility of controlling the size of the fœtal head by restricting the mother's diet in cases of contracted pelvis. In an article on mastitis in cows Mr. James Henderson divides the lesions commonly found into three classes: Tuberculous, purulent, and parenchymatous, and discusses very clearly the micro-organisms concerned and their bearing especially on epidemic diarrhœa in children.

**The Science and Art of Prescribing.** By E. H. Colbeck, M.D. (Cantab.), F.R.C.P., and Arnold Chaplin, M.D. (Cantab.), F.R.C.P. Second Edition. Pp. x., 198. London: Henry Kimpton. 1906.—An extremely useful text-book, which would with advantage be in the hands of every medical student and every medical man starting in practice. The opening chapters deal successively with the prescription—the rules for writing one and the analysis of a model prescription; incompatibility—chemical, pharmaceutical, and therapeutical; and the "Administration of Drugs." The chapter dealing with this last gives a comprehensive survey of mixtures, emulsions, pills, powders, tablets, gargles, lozenges, &c., together with a complete table showing all the drugs in general use, with their doses and best methods of exhibition. Part II. is an application of Part I., classifying the various ills to which man is heir, and showing under each the drugs particularly applicable and their best form of administration. The appendix strikes the right note in its opening sentence, in which the practice of prescribing

"patent" remedies of unknown composition is strongly deprecated. It also gives a list of the "quack" remedies of whose advertisements the Press is unfortunately teeming, with their approximate constitution, a very short perusal of which should convince every disciple of Æsculapius that with this little handbook and the B.P. he can finally discard "Jones' Liquor Albus," write his own prescription of known drugs, and feel sure that his patient is getting what his prescriber knows is the desirable medicament. Therefore the book should be bought and read.

**Gynæcological Diagnosis.** By ARTHUR E. GILES, M.D., B.Sc. Lond. Pp. x., 212. London: Baillière, Tindall and Cox. 1906.—In systematic works the disease is mentioned first and the detailed symptoms follow. This plan necessarily involves a preformed diagnosis before the subject can be followed. The author's method is the converse of this. He takes each of the leading symptoms usually met with in gynæcological diseases and argues from the general to the particular, so that a diagnosis is made from the most prominent symptoms of the disease as a starting-point. At the end of each division the various conditions leading to the diagnosis are set out in tabular form. The method necessarily involves some repetition, but it is ingeniously worked out; and though the idea is subject to the imperfections which necessarily attend a cut-and-dried didactic form, it is a praiseworthy attempt to elucidate a class of disease in which the practitioner may have had scant opportunities of acquiring such familiarity as is needful for the formation of an accurate diagnosis. With the reservation that we have mentioned, we can recommend the book as a sound and helpful handbook when used in association with a systematic work on gynæcology.

**Public Health Administration in Glasgow:** *A Memorial Volume of the Writings of James Burn Russell, B.A., M.D., LL.D.* Edited by A. K. CHALMERS, M.D. Pp. xxxii., 612. Glasgow: James Maclehose & Sons. 1905.—The student of Public Health owes a debt of gratitude to the editors of this delightful volume for rescuing from the oblivion of reports and magazines these admirable contributions to the literature of Public Health. Dr. Russell was no mere writer of perfunctory reports; his essays stand out as lucid studies of the inner meaning and practical possibilities of Public Health Administration. To our mind, the gem of the collection is the essay "Public Health and Social Problems," but each essay in the book is thoughtful and weighty. The task of editing the book has been very sympathetically carried out by Dr. Chalmers, and Sir W. T. Gairdner contributes a warm personal appreciation. The volume is well brought out, and contains a speaking likeness of Dr. Russell.

